# CA15.3 Serum Concentrations in Older Women with Infiltrating Ductal Carcinomas of the Breast

**DOI:** 10.3390/ijms151119870

**Published:** 2014-10-31

**Authors:** Álvaro Ruibal, Pablo Aguiar, María Carmen Del Río, María Elena Padín-Iruegas, José Ignacio Arias, Michel Herranz

**Affiliations:** 1 Molecular Imaging Group, F. Medicine, University of Santiago Compostela, R/de San Francisco, s/n, Santiago de Compostela 15782, Spain; E-Mails: alvaro.ruibal.morell@sergas.es (A.R.); michel.herranz.carnero@sergas.es (M.H.); 2 Nuclear Medicine Department, University Hospital Santiago Compostela (CHUS), R/Choupana, s/n, Santiago de Compostela 15706, Spain; 3 Fundación Tejerina, C/José Abascal, 40, Madrid 28003, Spain; 4 General Lab, H. Virgen Xunqueira, P/Pepe Sánchez, 7, Cee, A Coruña 15270, Spain; E-Mail: Maria.del.Carmen.Del.Rio.Garma@sergas.es; 5 Anatomía Humana Area, University of Vigo, Lagoas-Marcosende, s/n, Vigo 36310, Spain; E-Mail: mariaelena.padin.iruegas@sergas.es; 6 Surgery Department, Hospital of Monte Naranco, Av Fernández Vega, 107, Oviedo 33012, Spain; E-Mail: joseignacio.arias@sespa.princast.es

**Keywords:** breast cancer, ductal carcinoma, CA15.3, elderly

## Abstract

Breast cancer is currently becoming a disease of the elderly. We have studied the relation between CA 15.3 serum concentrations and clinical-pathological parameters in 69 women with IDC aged over 70 years (76.3 ± 4.2; range: 71–88; median 76). A group of 205 women with the same tumor but aged <70 years (62.8 ± 4.0; range: 55–70; median 63) was also considered for comparison. Tumor size, axillary lymph node involvement, distant metastasis and histological grade were taken account. Serum CA 15.3 was determined by luminescence assay. CA 15.3 serum concentrations ranged between 6 and 85 U/mL (median 22.9 U/mL), and were higher only in patients with greater (qualitative and quantitative; *p*: 0.041) tumor size. Our results show that in women with IDCs, and aged over 70 years, serum CA 15.3 serum concentrations are associated exclusively with a greater tumor size, being these findings different to those described in women with the same subtype of tumor considered as a whole or with lower age.

## 1. Introduction

The carbohydrate antigen 15.3 (CA 15.3) is a tumor marker originated from the epithelial mucin 1 gene, which is expressed at the luminal epithelial layer. It is used in patients with breast cancer, and its main clinical application lies in the monitoring of them in order to detect early recurrences and/or distant metastasis, as well as to know the effectiveness of a therapy [1,2,3]. Likewise, this marker can be useful with PET/CT to show tumor recurrence, especially when there is not any evidence by other imaging techniques [4]. In relation with CA 15.3, we know that its gene is under epigenetic control and has several biological functions such as cell adhesion, gastrointestinal mucosa infection defense, osmo-adaptation, proliferation, invasion, metastatic capacity, immune response and epithelial mesenchymal transition [5].

At the present, breast cancer is the most frequently diagnosed cancer and the leading cause of cancer death in females worldwide. Furthermore, breast cancer is becoming a disease of the elderly, due one of the most important risk factors for this tumor is the age. Women aged over 70 years having *in situ* ductal carcinomas usually receive less aggressive therapies and some groups do not observe differences in tumor size, hormone-dependence and other pathological tumoral features in relation to age. The 5 year percentage of local recurrence was lower (3%) in older patients than in women below 40 years (10%) [6]. Regarding infiltrating tumor, it is accepted that older women generally have a better prognosis and for this reason it has recently been considered that older women would be included in the mammography screening program in order to obtain a significant reduction in tumor size at diagnosis [7]. It is interesting to note that 15%–18% of breast tumors in older women are triple negative (ER-negative, PR-negative, ER2.negative) and their outcome seems to be better than in younger patients. Likewise, the therapy for these tumors in older women is less aggressive than in younger patients [8]. In women aged >75 years (mean age 80 years) comorbidities, pT (tumor size after pathological analysis) stage, metastases and hormone receptor status were independent prognostic factors for relative survival [9]. In Spain the trend in mortality due to breast cancer decreased from 1993 to 2007 and this trend continues at the present time. However, the mortality decreased in younger and middle-aged patients, but in older women remained unchanged [10].

For many years, CA 15.3 was used in clinical practice as a serum tumor marker, especially from breast origin [11], although it does not increase in early stages and in some local recurrence (20%–35% of positive results). Nevertheless, it is possible to see high levels in other malignant tumors, as well as in certain non-neoplastic diseases [12]. We know that higher preoperative CA 15.3 levels are associated with a worse survival and with the simultaneous determination of carcinoembryonic antigen (CEA) levels were independent prognostic factor after a multivariate analysis [13]. Also, CA 15.3 provides additional information to the common prognostic factors and should be considered in the adjuvant therapeutic algorithm [14]. Nevertheless, some groups disagree on the usefulness of CA 15.3 determination in asymptomatic patients, because it cannot improve their survival [15]. Some groups have described that CA 15.3 serum concentrations are associated with certain clinical and pathological features of the primary tumor such as age, size, lymph node involvement and distant metastasis, cellular proliferation, histological grade, HER2 expression, molecular subtype and perivascular invasion [13,14,16,17]. The relationship between CA 15.3 serum concentrations and hormone-dependence is less clear. Recently [18], our group have described higher CA 15.3 serum concentrations in postmenopausal women, tumors >2 cm, positive axillary involvement and distant metastasis. Likewise, we observed an inverse association between CA 15.3 serum concentrations and S-phase fraction, cell surface EGFR and cytosolic cathepsin D values [17]. In this work, we have focused on a patient group of older women with infiltrating ductal carcinomas of the breast, studying the behavior of serum CA 15.3 levels and their possible associations with some clinical and pathological parameters.

## 2. Results and Discussion

In the study group, CA 15.3 serum concentrations ranged between 6 and 85 U/mL with median of 22.9 U/mL. As seen in Table 1, those were higher only in patients with greater size (cut-off used: 2 cm; *p*: 0.041). There were not statistically significant differences when axillary lymph node involvement (N), distant metastasis (M) and histological grade (HGI *vs.* HGIII; HGII was excluded in order to obtain more precise conclusions) were considered. These results are different to those observed in patients with the same subtype of tumor but with an age range from 55 to 70 years.

Different clinical and pathological parameters analyzed according to global antigenic levels and a quantitative arbitrary upper normal level of 23 U/mL, which represents the median value of CA 15.3 in the whole group of patients, are shown in Table 2. It shows that also only the tumor size was higher (*p*: 0.018 and *p*: 0.041 respectively) in positive patients.

**Table 1:** Carbohydrate antigen 15.3 (CA15.3) concentrations in women aged >70 years with invasive ductal carcinomas of the breast and classified according to different clinical and histological parameters (CA15.3 concentrations in women aged <70 years are also shown).

	Number of Patients		Median (Range)		*p*-Value	
Parameter	>70 years	<70 years	>70 years	<70 years	>70 years	<70 years
N−	40	136	21 (6–45)	17 (1–57)	ns	0.006
N+	29	69	23 (7–54)	23 (6–191)		
M−	54	191	22 (6–55)	18 (4–162)	ns	0.001
M+	15	14	25 (10–86)	32 (11–17,340)		
HGI	9	38	24 (11–32)	16 (8–17,340)	ns	0.085
HGIII	23	64	20 (10–55)	20 (4–191)		
≤2 cm	35	142	20 (6–42)	18 (1–57)	0.041	0.014
>2 cm	34	63	25 (7–86)	23 (8–17,340)		

N: axillary lymph node involvement; M: distant metastasis; HG: histological grade; ns: no significant.

**Table 2:** Distribution of the different clinical and histological parameters in patients with invasive ductal carcinomas of the breast in women aged over 70 years and classified according to the positive cut-off (23 U/mL; 50th percentile of the whole subgroup).

	>23	≤23	*p*-Value
Parameter	Median (Range)	Median (Range)	
Size	2.5 (0.7–8)	2.0 (0.9–9)	0.018
	**Number of Patients**	**Number of Patients**	
>2 cm	20/32 (63%)	14/37 (38%)	0.041
N+	14/32 (44%)	16/37 (43%)	ns
N + 3	7/32 (22%)	12/37(32%)	ns
M+	8/32 (25%)	7/37 (19%)	ns
HGIII	9/32 (29%)	14/37 (38%)	ns

N: axillary lymph node involvement; M: distant metastasis; HG: histological grade; N + 3: more than 3 axillary lymph nodes involved.

Due to breast cancer in older women currently being a major social problem, in this work we have studied the CA 15.3 serum concentrations in women over 70 years having IDCs and their possible associations with some clinical and pathological parameters used in clinical practice. Our work was focused to study the existence of possible differences in CA 15.3 serum associations related to age. We have observed that marker serum concentrations did not differ significantly between the presence or absence of axillary lymph node involvement, distant metastasis and histological grade, being exclusively higher in tumors with higher size and in size >2 cm *vs.* ≤2 cm. These results seem to show that the associations of serum CA 15.3 serum concentrations in older women with other clinical and pathological parameters are different to those described in patients with the same tumor but considered as a whole. There were not observed the associations with lymph node involvement and distant metastasis and histological grade described in the literature [1,16,17]. The same happened when we defined a threshold of positivity to the marker in the 50th percentile value of the entire study group. Our findings were in agreement with Lumachi *et al.* [18] who illustrated in the subgroup of patients aged over 65 years who developed relapse, both CA 15.3 and CEA baseline serum concentrations were lower than in the subgroup of disease free patients. Although serum tumor markers may be useful during follow-up, their baseline levels are not useful in predicting relapse in elderly patients. Maybe tumor biology changes with age and markers synthesis and release is reduced and/or modified. This would be consistent with our previous results which showed a lower influence of hormone dependence (ER and PR) on different clinical and pathological parameters in women over 70 years relative to that found in younger women [19].

## 3. Experimental Section

### 3.1. Study Design

Tumor size, axillary lymph node involvement (N), distant metastasis (M), and histological grade (HG) were obtained from a patient group of older women with invasive ductal breast carcinomas in order to study their possible associations with CA 15.3 serum levels.

### 3.2. Subjects

The study group included 69 women with IDCs (other histological subtypes were excluded) aged over 70 years (76.3 ± 4.2; range: 71–88; median 76) who had undergone no prior treatment. All were studied at the Breast Cancer Unit of our hospital. Our study received the Ethical Committee approval from our hospital. The results were compared with those observed in 205 women with the same tumor but with an age ranged between 55 and 70 years (62.8 ± 4.0; range: 55–70; median 63).

### 3.3. Blood Samples and Methods

Serum samples were obtained 2–5 days before surgery, being patients fasted overnight. Serum samples were aliquoted and stored at −20 °C until they were assayed (<7 days). Serum CA 15.3 was determined using a luminescence assay (ECLIA-Elecsys 170, Roche, Basel, Switzerland), with two monoclonal antibodies (115D8 and DF3) and with a lower limit of sensitivity of 1 U/mL. Intraassay variation for a mean value of 93.5 U/mL was 2.9% and interassay variation coefficient for a mean value of 78.8 U/mL was 4.9%.

Tumor size, N, M, and HG were studied after the tumor resection in the Pathology Department.

### 3.4. Statistics Analysis

Data obtained were evaluated using the SPSS 15.0 software for Windows (SPSS, Chicago, IL, USA). Due to that they did not follow a normal distribution (Smirnoff-Kolmogrorof). CA 15.3 values were presented as range and 50th percentile value (median). We used the Chi square test with Yates correction, if necessary, for qualitative variables comparison and the Mann Whitney test for continuous ones. When it was necessary, Kruskal Wallis test was used for comparison of CA 15.3 with other parameters. A *p*-value <0.05 was considered as statistically significant.

## 4. Conclusions

Our results show that in women over 70 years and with breast IDC, serum CA 15.3 concentrations are associated exclusively with a greater tumor size, in contrast to the reported data regardless the age. We assume that the small sample size of the group of women over 70 years is a limitation of our study. Nevertheless, given the difficulty of obtaining data for older women (no published studies for women aged >70 years), we strongly believe that our work is still of interest as a preliminary result and it opens the door to further studies to reinforce our findings and their clinical impact for diagnosis and follow-up of breast IDC in elder women.
